# Mating Pheromone (Gamone 1) in *Blepharisma*: A Glycoprotein Responsible for Species Diversity in Unicellular Organisms (Alveolata, Ciliophora)

**DOI:** 10.3390/microorganisms12020299

**Published:** 2024-01-30

**Authors:** Mayumi Kobayashi, Mayumi Sugiura, Shoko Iwasaki, Naoyuki Iwabe, Terue Harumoto

**Affiliations:** 1 Division of Science, Graduate School of Humanities and Sciences, Nara Women’s University, Nara 630-8506, Japan; m.kobarun@gmail.com; 2 Research Group of Biological Sciences, Division of Natural Sciences, Nara Women’s University, Nara 630-8506, Japan; msugi@cc.nara-wu.ac.jp; 3 Biological Sciences Course, Graduate School of Humanities and Sciences, Nara Women’s University, Nara 630-8506, Japan; shokoi0903@gmail.com; 4 Department of Biophysics, Graduate School of Science, Kyoto University, Kyoto 606-8502, Japan; iwabe.naoyuki.7x@kyoto-u.ac.jp; 5 Center for Diversity and Inclusion, Nara Women’s University, Nara 630-8506, Japan

**Keywords:** ciliate, mating pheromone, reproductive isolation, species diversification, speciation

## Abstract

The genus *Blepharisma* (Alveolata, Ciliophora) is a unicellular organism distributed worldwide, even in extreme environments, and comprises numerous species. While usually proliferating through cell division, *Blepharisma* undergoes sexual reproduction (conjugation) when cells are moderately starved. Conjugation is initiated by mating pheromones (gamone 1 and gamone 2) secreted by complementary mating-type cells. Gamone 1, a glycoprotein, functions in a species-specific manner, while gamone 2, an amino acid derivative, is a common molecule across species. The specific function of gamone 1 suggests the possibility that mutations in gamone 1 might have led to reproductive isolation during the evolutionary process, triggering species diversification. In this study, by comparing the amino acid sequences of gamone 1 homologs from 15 strains (representing five species), we found that mutations resulting in distinct amino acid properties occur across species boundaries and are mainly concentrated at two specific regions within gamone 1. These mutations potentially alter the binding affinity of gamone 1 to its receptors, suggesting their effect in causing reproductive isolation. The interspecies artificial conjugation conducted previously and the molecular phylogenetic tree constructed using the gamone 1 homolog sequences in this study provide insights into the speciation process within the genus *Blepharisma*.

## 1. Introduction

*Blepharisma* (Alveolata, Ciliophora) is a genus widely distributed worldwide [1,2], even in extreme environments [3,4], that exhibits highly diverse ranges of cell sizes and nuclear morphologies [1]. *Blepharisma* species are classified mainly based on morphological characteristics and SSU rRNA gene sequences [5,6,7,8]. So far, dozens of species (morphospecies) within this genus have been reported, and the number of known species is continually increasing [6,8]. While some of these evolutionary relationships are not monophyletic and show substantial molecular divergence, the majority of them belong to a single clade [9]. However, in phylogenetic trees based on the SSU rRNA gene, there are few discernible differences, making the relationships between species difficult to understand. These species are divided into at least four groups of megakaryotypes (I–IV designated as Mk1–Mk4, here) based on the morphology of the macronucleus (Mk1: compact macronucleus; Mk2: binodal macronucleus; Mk3: multinodal macronucleus; and Mk4: filiform macronucleus), and several species belong to the Mk1 through Mk4 groups [10]. However, it remains unclear how these species and megakaryotypes evolved.

The mechanism underlying species diversification is a fundamental problem in evolutionary biology. Pre- and postzygotic reproductive isolation mechanisms are involved in the process of diversification. For insects and some vertebrates, the specificity of pheromones and receptors determines the reproductive group [11,12,13]. Mutational alterations of the mating pheromone system affect male/female recognition, resulting in prezygotic isolation. It is thought that such changes in pheromonal recognition generate novel reproductive groups, thereby triggering speciation [11,13]. In budding yeast, the modification of the sexual pheromone structure led to the creation of yeast strains carrying inactive pheromones that were no longer recognizable by the wild type, resulting in artificial reproductive isolation [14]. Conversely, by introducing random mutations into receptor genes and generating a large population (approx. 600,000 individuals) with mutant receptors, individuals capable of recognizing the altered pheromones were selected. Combinations of mutated pheromones and mutant receptors displayed heightened mating activity [14]. This experimental evidence demonstrates that mutations in sexual pheromones can induce reproductive isolation, potentially leading to the emergence of new species. It strongly suggests that mutations occurring in sexual pheromones might serve as a contributing factor in generating reproductive isolation, thereby fostering the birth of new species.

*Blepharisma* undergoes sexual reproduction (conjugation), resulting in offspring with a new genetic composition. Conjugation usually occurs through the interaction between complementary mating-type cells (I and II) via mating pheromones called gamones [15,16,17]. Under food-deprived conditions, mating type I cells secrete gamone 1 (blepharmone) [15,18] while mating type II cells secrete gamone 2 (blepharismone) [15,19]. These pheromones stimulate each other’s cells, which then form mating pairs. Gamone 1 is a glycoprotein with N-linked oligosaccharides [18,20,21] and is encoded by the gamone 1 gene [21]. Gamone 1 is a glycoprotein possessing four attachment sites with N-linked oligosaccharides [20,21]: glucosamine 4 and mannose 3. Gamone 2 is an entirely different substance, an amino acid derivative identified as 3-(2′-formylamino-5′-hydroxybenzoyl) lactate [19]. Gamone 1 reportedly has a species-specific effect in the induction of conjugation, and gamone 2 is common among *Blepharisma* species [22,23,24]. The phenomenon of entirely different substances inducing conjugation in type I and type II is intriguing, as there are no examples in other ciliates.

As gamones are molecules implicated in sexual reproduction as mating pheromones of *Blepharisma*, their potential involvement in species diversification is also intriguing. Since gamone 2 is common among species, it seems unlikely that mutations in gamone 2 contribute to speciation. On the contrary, gamone 1, being a glycoprotein, exerts species-specific effects. It thus seems worthwhile to investigate the possibility that gene mutations in gamone 1 influence speciation.

In this study, we hypothesize that the glycoprotein gamone 1 played a role in the species diversification of *Blepharisma*. Initially, we examined whether oligosaccharides are involved in the activity of gamone 1. Next, by comparing the amino acid sequences of gamone 1 between species, we explored the potential influence of mutations in gamone 1 on speciation. Additionally, we discussed the morphological species (morphospecies) and biological species within *Blepharisma*.

## 2. Materials and Methods

### 2.1. Strains and Culture

The strains used in this study are listed in Table 1. The 7 newly obtained and characterized strains were CCAP1607/1 (*B. americanum*), SM-III (*B. americanum*), KR-III *(B. americanum*), SDT0-III (*B. musculus*), SG1-III (*B. musculus*), KG-IV (*B. japonicum*), and WK-IV (*B. stoltei*). Strain CCAP1607/1 was purchased from the Culture Collection of Algae & Protozoa (UK). All stocks were cultivated and maintained in our laboratory. Cells were cultured at 25 °C in WGP (Wheat Grass Powder, Pines) medium [21] or diluted fresh lettuce juice medium inoculated with *Enterobacter aerogenes* 2 days before use. When the cells reached the stationary phase, they were concentrated using mild centrifugation, washed with SMB (Synthetic Medium for *Blepharisma*: physiologically balanced solution, SMB III medium without EDTA) [16], and suspended in SMB at 1500–2000 cells/mL density.

### 2.2. Preparation of Cell-Free Fluid (CFF)

To obtain CFF containing gamone 1, moderately starved type I cells were incubated at 25 °C for 2 days. The cells were then removed from the suspension using mild centrifugation, and the remaining CFF was filtered through a nylon net (mesh size 1 × 1 µm) and then through a 0.20 μm DISMIC-25 cs filter. The prepared CFF was designated as CFF1.

### 2.3. Investigation on the Presence of Oligosaccharides in Gamone 1 of Two Species

#### 2.3.1. Treatment of Samples Using Con-A Beads, and Preparation of Flow-Through Fraction and Elution Fraction

The samples were purified using agarose-bound Con-A (Vector laboratories, Newark, CA, USA). Initially, the buffer of Con-A beads was exchanged with SMB and stirred with CFF (1 h, 4 °C). After centrifugation, a portion of the supernatant was collected as the flow-through fraction (FT). Following 3 washes, the mixture was left to settle after the addition of elution buffer (0.5 M methyl-D-mannopyranoside in SMB). Upon centrifugation, the supernatant was collected as elution fraction (E).

#### 2.3.2. SDS-PAGE and Western Blotting

The sample was concentrated to 2–10 times its original concentration using Amicon Ultra Centrifugal Filter, 10 kDa (Merck Millipore, Burlington, MA, USA). The sample was mixed with an equal volume of double the concentration of sample buffer (0.1 M Tris-HCl (pH 6.8), 4% SDS, 12% β-mercaptoethanol, 20% glycerol, and bromophenol blue) and subjected to heat treatment (95 °C, 5 min). SDS-PAGE was conducted using a 15% running gel and a 4.75% stacking gel subjected to electrophoresis at 40 mA/gel for 3 h, followed by silver staining. Western blotting was carried out using a semi-dry method, employing PVDF membrane (Clear Blot membrane-P, AE-6666, ATTO, Tokyo, Japan). The Horizon blot (AE-6677, ATTO) was utilized to transfer at 2 mA/cm^2^ for 60 min. Blocking was conducted using Blocking One (Nacalai tesque, Kyoto, Japan). G1_P6 antibody (a peptide antibody recognizing the C-terminus of gamone 1 in *B. japonicum*, Thermo Scientific, Waltham, MA, USA) [25] was used as the primary antibody. The detection of the signal was achieved using Amersham ECL Plus Western blotting detection reagents (GE healthcare, Chicago, IL, USA), and exposure was performed on X-ray film.

### 2.4. Preparation and Bioassay of Gamone 1 with and without N-Linked Oligosaccharides

#### 2.4.1. Cleavage of N-Linked Oligosaccharides by Glycopeptidase F

**Figure 1:**
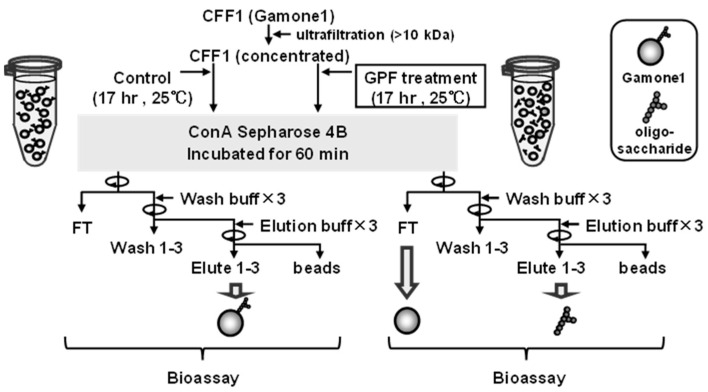
Procedure for the digestion of gamone 1 with glycopeptidase F (GPF) and bioassay of gamone 1 with and without oligosaccharides for conjugation-inducing activity. Gamone 1 activity (U/mL) was measured as described in Materials and Methods. FT: flow-through fraction; Wash: wash fraction; Elute: elution fraction.

CFF1 containing gamone 1 was first filtered through Amicon Ultra-4 or Amicon Utra-15 (Merck Millipore, Burlington, MA, USA) to concentrate gamone 1. After exploring various conditions, the optimal conditions for the glycopeptidase F (Takara Bio, Kusatsu, Japan) treatment involved the addition of 0.16 volumes of SMB as a buffer and 0.1 volumes of enzyme per 1 volume of CFF, followed by incubation at 25 °C for 17 h (Figure 1). Post enzyme treatment, the cleavage of gamone 1 oligosaccharide was confirmed using SDS-PAGE.

#### 2.4.2. Preparation of Gamone 1 with and without N-Linked Oligosaccharides

As shown in Figure 1, after the cleavage of N-linked oligosaccharides, samples were incubated with Con-A Sepharose 4B (GE healthcare) for 60 min. After centrifugation, a portion of the supernatant was collected as the flow-through fraction (FT). Following 3 washes (wash fraction W1–W3), the mixture was left to settle after the addition of elution buffer (0.5 M methyl-D-mannopyranoside in SMB). Upon centrifugation for 3 times, each supernatant was collected as elution fraction (E1–E3). Control samples were also prepared in the same way without the treatment of glycopeptidase F.

#### 2.4.3. Bioassay of Conjugation-Inducing Activity

The measurement of the activity of gamone 1 was performed using the unit method described by Miyake [16,18]. A 1 mL sample was sequentially diluted by a factor of two, creating dilutions up to 2^16^. Subsequently, 500 μL of tester cell suspension was added to each well of this serial two-fold dilution series. The cells were observed over time. The tester cells (Type II) used for the bioassay were collected and adjusted to a concentration of 1500–2000 cells/mL in SMB, 1–3 days prior to the assay. The formation of pairs reached a maximum generally within 2–3 h. If pairs were observed even up to a dilution of 2^N^, the sample was considered to possess an activity of 2^N+1^ [16,18]. In the unit method, one unit is defined as the amount of gamone that can induce the formation of one pair among 750–1000 tester cells suspended in 1 mL of the sample [16,18].

### 2.5. Extraction of Genomic DNA

Cultured cells were collected using centrifugation. To eliminate the red pigment, blepharismin, the cell suspension was subjected to cold shock by placing it on ice. The cells were collected using centrifugation, and twice the volume of lysing solution (20 mM Tris-HCl, 2.5% SDS, 50 mM EDTA, pH 9.5) [26,27] was added to the cell suspension and incubated with gentle agitation (65 °C, 15 min). An equal amount of Tris-EDTA saturated phenol was then added, and the mixture was treated for 15 min. The mixture was centrifuged to recover the aqueous phase. After ethanol precipitation, the DNA was resuspended in TE buffer and treated with RNase (100 µg/mL). Subsequently, ethanol precipitation was performed, and the DNA was resuspended in TE or sterile distilled water.

### 2.6. Isolation of Gamone 1 Homologs and Sequencing

The PCR for amplifying the gamone 1 homologs using genomic DNA as the template was conducted as follows. After the initial denaturation at 94 °C for 10 min, 30 cycles of denaturation at 94 °C for 30 s, annealing at 50 °C for 1 min, and extension at 72 °C for 2 min were performed with Ampli*Taq* Gold (Applied Biosystems, Waltham, MA, USA). When PCR was performed using a single cell, the cells were initially treated at 98 °C for 10 min, and then denatured at 94 °C for 5 min. Subsequently, 35 cycles of denaturation at 94 °C for 30 s, annealing at 49 °C for 30 s, and extension at 68 °C 2 min were performed with KOD FX (Toyobo, Osaka, Japan) or KOD FX Neo (Toyobo). Primer combinations Gm-30FW (5′-CTATTTAAGGCCGATCTTC-3′) and Gm1138Rv (5′-GTGATTTTGCCTTCAGAGTTC-3′), or Gm-30FW and Gm1336Rv (5′-TTCAAAGACGTCTGGCTTAG-3′), all designed based on the sequence of the gamone 1 gene from the R1072 strain, were used. The gamone 1 homologs were cloned and purified using the TOPO TA Cloning kit (Invitrogen, Waltham, MA, USA), followed by sequencing (ABI3500, Applied Biosystems).

### 2.7. Hydrophobic Profile Search and Molecular Phylogenetic Analyses

A hydrophobic profile search was conducted using GENETYX Ver. 7 (Genetyx Corporation). The amino acid sequences of gamone 1 homologs were aligned using MAFFT [28], and then manually inspected and improved. Excluding positions with alignment gaps and ambiguous alignment regions of weak sequence similarity, 197 amino acid positions in total were used for the molecular phylogenetic analyses of 10 strains (5 species) of *Blepharisma* and a homolog of *Stentor coeruleus* as an outgroup. The maximum likelihood (ML) tree was constructed using RAxML version 8 [29], a ML tree search program, using WAG (Whelan and Goldman) [30] and Yang’s discrete gamma model [31] with an optimized shape parameter alpha. Bootstrap probabilities for the branches of the ML tree were obtained through 200 bootstrap resamplings [32,33]. Statistical significance for ML and other possible tree topologies were evaluated by using approximately unbiased (AU) [34], Kishino–Hasegawa (KH) [33], and Shimodaira–Hasegawa (SH) [35] tests. The accession numbers of the amino acid sequences of gamone 1 and homologs are as follows: strain EN-II (*B. undulans*, acc. no. AB920333), SDT2-II (*B. undulans*, acc. no. AB920337), K78 (*B. undulans*, acc. no. AB920335), SDT1-II (*B. undulans*, acc. no. AB920336), YC-IV (*B. japonicum*, acc. no. AB920338), ATCC30299 (*B. stoltei*, acc. no. AB920332), HT-IV (*B. stoltei*, acc. no. AB920334), CCAP1607/1 (*B. americanum*, acc.no. LC795784), SDT0-III (*B. musculus*, acc.no. LC795785), SG1-III (*B. musculus*, acc.no. LC795786), SM-III (*B. americanum*, acc.no. LC795787), KG-IV (*B. japonicum*, acc.no. LC795788), WK-IV (*B. stoltei*, acc.no. LC795789), KR-III (*B. americanum*, acc.no. LC795790), and *Stentor coeruleus* (acc. no. OMJ76159), aligned with prepro-gamone 1 of strain R1072 (*B. japonicum,* acc. no. AB056696).

## 3. Results

### 3.1. Investigation into the Involvement of Oligosaccharides in the Conjugation-Inducing Activity

In *B. japonicum* of Mk4, it has been reported that N-linked oligosaccharides are attached to gamone 1 [21]. It was revealed that gamone 1 of *B. japonicum* had an affinity to Con-A but no affinity to lentil lectin or wheat germ agglutinin [21]. These results suggested that the oligosaccharide of gamone 1 is of the N-linked type without fucose modification in α1,6 linkage to the innermost N-acetylglucosamine residue.

**Figure 2:**
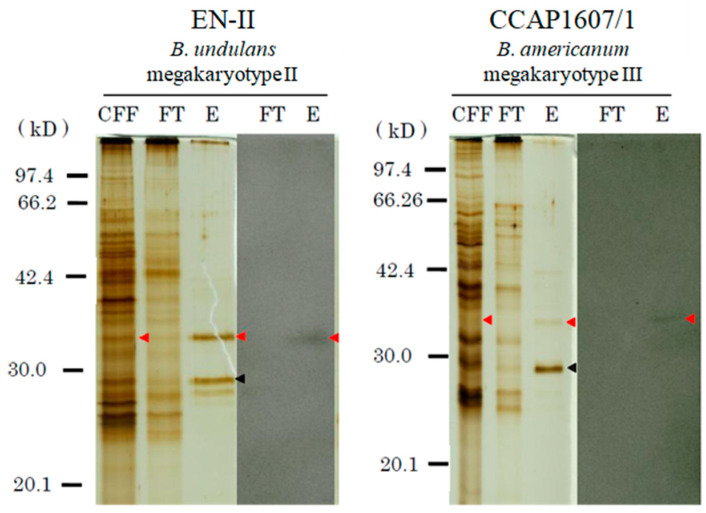
N-linked oligosaccharides in gamone 1 of stocks EN-II (mating type I, Mk-2) and CCAP1607/1 (mating type I, Mk-3). CFF obtained from each stock was mixed with agarose-bound Con-A beads. The fractions that bound to Con-A (E) and those that did not (FT) were separated and subjected to SDS-PAGE. In each stock, the three lanes from the left indicate CFF (cell-free fluid), FT (flow-through fraction), and E (elution fraction), respectively, while the right two lanes display the Western blotting results for FT and E. The red and black arrowheads show gamone 1 and Con-A, respectively. The gamone 1 band was detected only in the lane E.

In the present study, we investigated whether gamone 1 in species from Mk2 and Mk3, apart from Mk4, also possesses N-linked oligosaccharides. Cell-free fluid from stocks EN-II (mating type I, Mk2) and CCAP1607/1 (mating type I, Mk3) was prepared as described in the Materials and Methods. We added each CFF1 obtained from the stocks to agarose-bound Con-A beads, stirred the mixture, and then collected the supernatant as the flow-through fraction (FT) through centrifugation. After washing the beads, we added a buffer to elute the bound molecules. Centrifugation was carried out to collect the elution fraction (E). We subjected these fractions to SDS-PAGE and performed Western blotting. The results are shown in Figure 2. Only the band corresponding to gamone 1 was detected in the elution fraction in each stock, but it was not present in the flow-through fraction. This indicates that gamone 1 from these two strains also binds to the Con-A beads. Further experiments are needed, but there appears to be no contradiction in considering the presence of N-linked oligosaccharides with an affinity for Con-A in these two strains belonging to Mk2 and Mk3.

**Figure 3:**
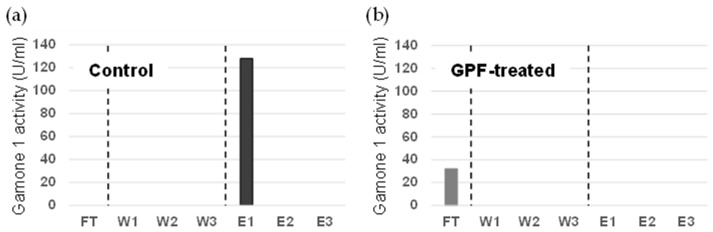
Bioassay of gamone 1 with and without oligosaccharides for conjugation-inducing activity. Gamone 1 activity (U/mL) was measured as described in Materials and Methods. FT: flow-through fraction; W: wash fraction; E: elution fraction. Conjugation-inducing activity of gamone 1 with (**a**) and without (**b**) oligosaccharides.

Next, we examined the extent to which oligosaccharides are involved in the conjugation-inducing activity by cleaving them with glycopeptidase F. As shown in Figure 3, even after the oligosaccharides were cleaved, the activity was not completely lost. In control not treated with GPF, gamone 1 with oligosaccharides was recovered in the elution fraction, exhibiting conjugation-inducing activity (Figure 3a). In GPF-treated samples, gamone 1 lacking oligosaccharides was recovered in the flow-through (FT) fraction, with a slight retention of conjugation-inducing activity (Figure 3b). Therefore, while it remains plausible that oligosaccharides are somehow involved in maintaining the activity, they may not be essential for it. Moreover, since oligosaccharides have a simple structure [20,21], it is unlikely that differences in species are attributable to differences in oligosaccharides. We hypothesized that the diversity in the amino acid sequence of gamone 1 might be more critical than its oligosaccharides and might be related to the differences between species.

### 3.2. Comparison of Amino Acids Sequences of Gamone 1 among Species Belonging to Mk2, Mk3, and Mk4

#### 3.2.1. Gamone 1 Genes in Mating Type I and II, and Newly Isolated Gamone 1 Homologs

Recent results of sequencing the gamone 1 gene between mating types I and II of *B. japonicum* revealed that both types harbor the gamone 1 gene in their genomes. Additionally, the type can be changed from type I to type II (or from II to I) after long cultivation. This suggests that the expression of mating type I or II is regulated at the expression level rather than at the genetic level (deletion, etc.). For these reasons, in this study, the gamone 1 homologs were isolated regardless of the mating type.

We previously reported the amino acid sequences of gamone 1 for eight strains (three species) [21,23]. In the present study, we newly isolated and determined the sequences of the gamone 1 homologous genes for three strains (CCAP1607/1, SM-III, and KR-III) of *B. americanum* (Mk3), two strains (SDT0-III and SG1-III) of *B. musculus* (Mk3), one strain (KG-IV) of *B. japonicum* (Mk4), and one strain (WK-IV) of *B. stoltei* (Mk4). Ultimately, we compared the amino acid sequences among a total of fifteen strains (five species): four strains of *B. undulans* (Mk2), three strains of *B. americanum* (Mk3) and two strains of *B. musculus* (Mk3), and three strains each of *B. japonicum* (Mk4) and *B. stoltei* (Mk4).

#### 3.2.2. ORF Length, Number of Estimated Oligosaccharide Attachment Sites, and Stop Codon in Gamone 1 Homologs of 15 Strains

The ORF lengths of gamone 1 homologs shows in *B. undulans*, *B. americanum*, *B. musculus*, *B. japonicum*, and *B. stoltei* are 305, 307, 304, 305, and 305 amino acids, respectively. So, the ORF length varies slightly among species but demonstrates a consistent trend within the same species. Strains EN-II, SDT2-II, K78, SDT1-II, CCAP1607/1, SM-III, KR-III, SDT0-III, SG1-III, and R1072 each had four oligosaccharide attachment sites; strains KG-IV, HT-IV, and WK-IV each had five, and strains YC-IV and ATCC30299 each had six. The number of attachment sites did not vary according to species or megakaryotypes. The stop codons for gamone 1 homologs are all UAA. While UGA encodes tryptophan in *Blepharisma* [36,37], none of the gamone 1 homologs contained UGA in ORF.

#### 3.2.3. Comparative Analysis of Gamone 1 Homologs and Amino Acid Variability among Species

As shown in Table 2, we investigated the homology of gamone 1 among different species, focusing on the similarity within gamone 1 homologs (67–100%). Within the same species and the same megakaryotypes, relatively higher homology was observed. In particular, the homology between *B. japonicum* and *B. stoltei* was notably high, ranging predominantly from 87% to over 95%. Conversely, only low similarity (67–76%) was observed between strains belonging to each of Mk2, Mk3, and Mk4, as well as between *B. americanum* and *B. musculus* within Mk3.

**Figure 4:**
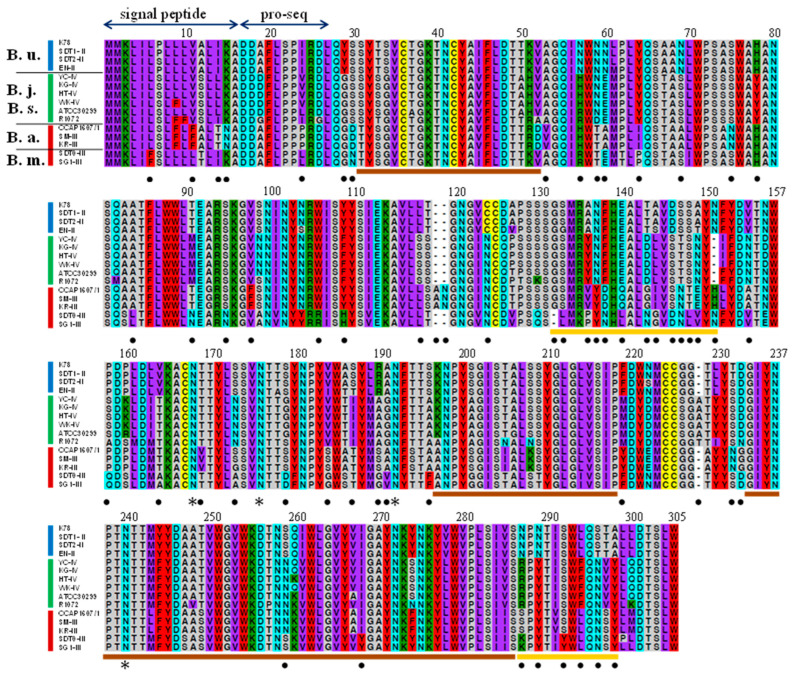
The amino acid sequence of gamone 1. Amino acids with similar characteristics have been categorized into six distinct groups, each highlighted in a different color [38]. B.u.: *B. undulans*; B.j.: *B. japonicum*; B.s.: *B. stoltei*; B.a.: *B. americanum*; B.m.: *B. musculus*. *: presumptive N-linked oligosaccharide attachment site. Filled circles indicate sites where mutations have occurred, leading to amino acids with different properties across species boundaries.

Gamone 1 exhibited regions with both minimal and high variability across species. Considering the properties of amino acids, we categorized amino acids with similar characteristics, such as size and hydrophobicity/hydrophilicity, into six distinct groups and color-coded them according to Miyata et al. [38] (Figure 4). Filled circles indicate sites where mutations have occurred, leading to amino acids with different properties across species boundaries. The analysis of the entire amino acid sequence of gamone 1 revealed three regions (31–51, 197–218, and 234–286) with minimal interspecies variability (shown by brown lines), while two regions (132–151 and 287–298) showed a concentration of filled circles (shown by yellow lines), representing sites with a higher occurrence of mutations that result in amino acids with differing properties across species boundaries. For example, at amino acid position 135, variations are A (*B. undulans*), Y (*B. japonicum* and *B. stoltei*), V (*B. americanum*), and P (*B. musculus*); at 142, T (*B. undulans*), D (*B. japonicum* and *B. stoltei*), G (*B. americanum*), and N (*B. musculus*); and at 287, N (*B. undulans*), R (*B. japonicum* and *B. stoltei*), S (*B. americanum*), and K (*B. musculus*). In these and other positions, it is noticeable that amino acids with different properties occur across species boundaries.

#### 3.2.4. Hydrophobicity Profiling and Estimated Sites for N-Linked Oligosaccharides

Hydrophobicity profiling indicated that the signal sequence and putative proline-rich regions displayed higher hydrophobicity. The region spanning 132–151 encompassed both hydrophobic and hydrophilic domains, whereas the region spanning 287–298 exhibited a higher tendency toward hydrophobicity. Remarkably, the hydrophobicity profiles showed minimal variance among species.

There exist four to six sites for binding N-linked oligosaccharides (of which three are common), yet within the concentrated areas of amino acid mutations across all the species, no binding sites for N-linked oligosaccharides were identified.

#### 3.2.5. Molecular Phylogenetic Tree of Gamone 1

**Figure 5:**
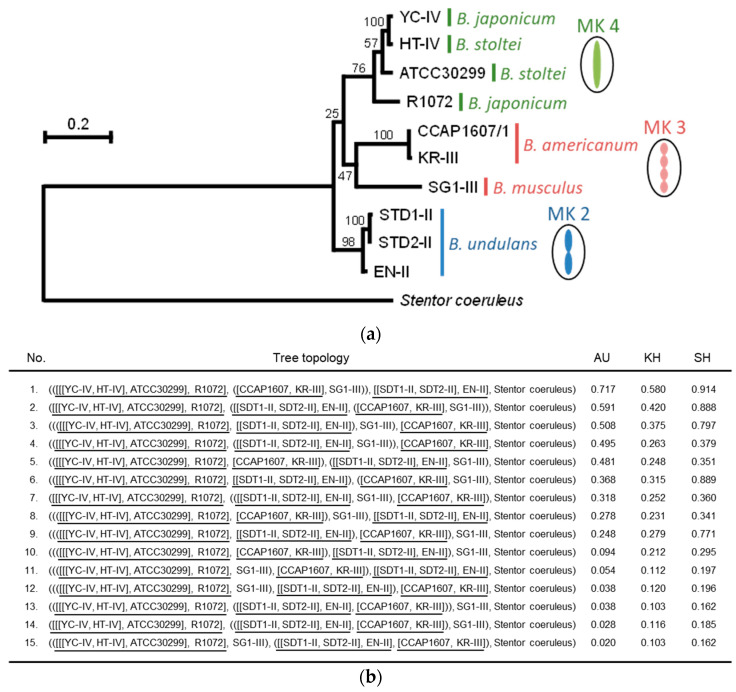
Detailed analysis of phylogenetic relationship among species of *Blepharisma*. (**a**) Molecular phylogenetic tree of gamone 1 homologs in 10 strains (five species) of *Blepharisma* with a homolog of *Stentor coeruleus* as an outgroup inferred using the maximum likelihood (ML) method. The ML tree of gamone 1 was constructed by using WAG [30] and Yang’s discrete gamma model [31] with an optimized shape parameter alpha (1.28). The numbers at each branch are bootstrap probabilities [32,33]. Megakaryotypes (Mk) are shown in different colors (green: Mk4; red: Mk3; and blue: Mk2). (**b**) ML and 14 other tree topologies of 11 gamone 1 homologs. Partial tree topologies denoted by [[[YC-IV, HT-IV], ATCC30299], R1072], [[SDT1-II, SDT2-II], EN-II], and [CCAP1607, KR-III] were fixed in this analysis because these topologies were contained in the ML tree of (**a**) and supported by relatively high bootstrap probabilities. Fixed partial topologies were underlined to make them easier to see. All 15 tree topologies for five OTUs, [[[YC-IV, HT-IV], ATCC30299], R1072], [[SDT1-II, SDT2-II], EN-II], [CCAP1607, KR-III], SG1-III, and *Stentor coeruleus*, were evaluated by using AU [34], KH [33], and SH [35] tests. The ML (tree number 1) and 10 other topologies (tree number 2 to 11) were not rejected by the AU test at the 5% level of significance. The values of the KH and SH tests are also represented in the list.

The mutations into different amino acids with distinct properties occur across species boundaries in gamone 1, which led us to consider these mutations as potential factors contributing to species diversification. To speculate on the evolutionary path of species diversification, a molecular phylogenetic tree was subsequently constructed (Figure 5), employing the maximum likelihood (ML) method using 10 strains (five species) of *Blepharisma*, with gamone 1 homologous sequence of *Stentor coeruleus* as an outgroup. Figure 5a represents the ML tree inferred using RAxML, a ML tree search program, using WAG and Yang’s discrete gamma model. Statistical significance for the ML and other possible tree topologies were evaluated by using AU, KH, and SH tests as shown in Figure 5b. Although the values of the AU test may indicate that many tree topologies were not statistically rejected, the ML tree (Figure 5a) suggests that the strains belonging to the same megakyaryotype were clustered, and Mk3 and Mk4 appeared more closely related, while Mk2 displayed a more distant relationship.

## 4. Discussion

### 4.1. Amino Acid Alterations of Gamone 1 and Possible Sites Responsible for Species Diversification

The present study revealed that the conjugation-inducing activities of gamone 1 persist even after the removal of oligosaccharides through treatment with glycopeptidase F (Figure 3). Also, considering the limited diversity in oligosaccharides, which appears insufficient to account for the species-specific effect of gamone 1, it is plausible to consider that the amino acid sequences contribute more to species-specific activity than their oligosaccharides.

The present study also revealed the sequences of gamone 1 homologs of seven new strains in addition to the previously sequenced eight strains limited to three species [23]. The amino acid sequences of gamone 1 homologs in five species exhibit regions with low variation as well as highly mutable regions among species (Figure 4). Notably, within the highly mutable regions, variations in amino acid sequences have emerged across species boundaries. Therefore, it is reasonable to suggest that the occurrence of mutations in these regions might have played a role in species diversification. Considering the functional locations of these mutations within the molecule of gamone 1, which is a globular protein binding to putative receptors on type II cells to facilitate signal transduction into the cell, if reproductive isolation arose due to gamone 1 mutations, the most probable site would be the region responsible for binding with the gamone 1 receptor. The two regions (132–150 and 287–298) exhibit a concentration of mutations leading to amino acids conferring distinct properties across species (Figure 4). Mutations within these regions may have led to alterations in the three-dimensional overall structure of gamone 1. Consequently, these changes could have affected the binding ability to gamone 1 receptors, resulting in the establishment of reproductive isolation. Subsequent mutations in the gamone 1 receptor of type II cells, enabling the recognition of the mutated gamone 1, could potentially give rise to a new combination of mutated gamone 1 and its receptor. This possibility suggests the emergence of a new species from an ancestral one. Such examples have been experimentally demonstrated in budding yeast [14]. The modification of the sexual pheromone created yeast strains with inactive pheromones that were no longer recognizable by the wild type. Introducing mutations into receptor genes generated individuals capable of recognizing the altered pheromones [14]. This experimental evidence demonstrates that mutations occurring in sexual pheromones may contribute as a factor in generating reproductive isolation. There is a significant possibility that the birth of a new species could also occur in *Blepharisma*.

### 4.2. Three Steps for the Completion of Conjugation and Establishment of the Steps during Species Diversification

Our research on interspecies conjugation revealed at least three essential steps for completion: (1) the gamone 1-mediated step (inducing mating pairs in type II cells), (2) assessing the formation of mating pairs by activated cells, and (3) producing offspring [23,24]. Essentially, gamone 1 exhibits species specificity; even within the same megakaryotype, it does not function across species (e.g., *B. americanum* and *B. musculus*, both belonging to Mk3, do not interact with each other) [22,23,24]. This implies that reproductive isolation essentially occurs at Step 1. However, a situation might occur where different species cohabit the same location, suggesting the potential for type II cells activated by the same species of gamone 1 to encounter activated type I cells and form mating pairs. We have reported on interspecies mating pair formation among artificially activated cells [23,24]. Yet, even when cells treated with gamone 1 of the same species are brought together, interspecies mating pairs are scarcely formed (e.g., between *B. undulans* (Mk2) and *B. japonicum* (Mk4), and between *B. undulans* (Mk2) and *B. americanum* (Mk3)), while in some cases, interspecies mating pairs are formed (e.g., between *B. americanum* (Mk3) and *B. japonicum* (Mk4) or between *B. americanum* (Mk3) and *B. stoltei* (Mk4). This indicates that while interspecies pair formation is feasible between certain species, it remains unattainable between others at Step 2. Moreover, even in cases where interspecies mating pairs between *B. americanum* (Mk3) and *B. stoltei* (Mk4) occur, no offspring are produced between species (i.e., infertility) [24]. In this case, while Step 1 and Step 2 were successfully passed, Step 3 was not.

The phylogenetic relationships of the species in *Blepharisma* are considered to follow a sequence of closeness. This sequence begins with a stage where, in the same species, gamone 1 functions, enabling the formation of mating pairs and offspring, successfully clearing Steps 1–3. This stage is considered the closest. It is followed by a stage where artificial mating pairs are formed but fail to produce offspring, with Step 3 remaining uncleared. Next is a stage where, even under artificial activation, mating pairs fail to form, failing to clear Steps 2–3. Finally, in progressively distant relationships, there is a stage where gamone 1 no longer functions, resulting in the failure to clear Step 1.

A consideration of the results obtained so far leads to the following speculation. The evolutionary process of speciation in *Blepharisma* appears to progress sequentially from stages where, within the same species, gamone 1 functions, enabling mating pairs to form and offspring to arise (successfully clearing Step 3). This succession is followed by a stage where artificial mating pairs are formed but fail to produce offspring (Step 3 remains uncleared), as observed between *B. americanum* (Mk3) and *B. stoltei* (Mk4). Subsequently, there is another stage where mating pairs can no longer be formed (failing to clear Step 2) even when artificially induced, as seen between *B. undulans* (Mk2) and *B. japonicum* (Mk4), as well as between *B. undulans* (Mk2) and *B. americanum* (Mk3). In this manner, the same species gradually diverge into more distant relationships.

This is further supported by the molecular phylogenetic tree based on the amino acid sequences of gamone 1 (Figure 5). The species in which artificial activation induces mating pair formation but does not yield offspring, such as *B. americanum* (Mk3) and *B. stoltei* (Mk4), are positioned closer in the phylogenetic tree. Conversely, species in which artificial activation fails to induce mating pair formation, such as *B. undulans* (Mk2) and *B. japonicum* (Mk4) or *B. undulans* (Mk2) and *B. americanum* (Mk3), are positioned further apart in the phylogenetic tree (Figure 5).

This tree also indicates the possible evolutionary path of diversification in the genus *Blepharisma*. The molecular mechanisms behind Steps 2 and 3 remain unclear. However, in Step 1, as discussed above, mutations in gamone 1 possibly induce conformational change and result in the inability to bind for gamone 1 receptor, leading to the loss of gamone 1 function, and promoting the reproductive isolation. Subsequent mutations affect factors involved in mating pair formation, leading to an inability to form mating pairs, even under the artificial cell activation. Next, even when artificial mating pairs are formed, mutations in various factors affecting offspring survival prevent their production. As reproductive isolation progresses, other mutations occur, causing significant nuclear morphological changes and contributing to the diversification of species observed today. In essence, it is postulated that the gradual establishment of barrier steps in the conjugation process within populations isolated by gamone 1 ultimately leads to diversification, and possibly speciation, in *Blepharisma*.

### 4.3. Species Problem in Paramecium and Blepharisma

The species concept in ciliates has been debated since the times of Sonneborn [39,40]. The *Paramecium aurelia* group, whose members are morphologically indistinguishable, was historically composed of 14 syngens in which conjugation did not occur. However, beyond the absence of offspring production within syngens, molecular phylogenetic analysis revealed distinct molecular differences among the syngens. It was consequently deemed appropriate to consider each syngen as a distinct species rather than a syngen, resulting in the classification of the *P. aurelia* complex into 14 biological species (i.e., sibling species) [41]. Afterward, a species designated as *P. sonneborni* was discovered, and currently there are 15 species in the *P. aurelia* complex [42]. In contrast, *Paramecium caudatum* consists of 16 known syngens that are morphologically indistinct from each other, yet reproductively isolated. However, deliberate intersyngen crosses produced viable offspring, indicating fertility between different syngens [43,44,45]. Hence, while reproductive isolation occurs at the early stages of conjugation between distinct syngens, the molecular elements involved in mating pair formation are assumed to be common. As viable offspring are produced, the differences between syngens have not yet reached the level of distinct species (biological species). Therefore, it is appropriate to use the term ‘syngens’ to describe the subdivisions within *P. caudatum* rather than categorizing them as biological species.

The genus *Blepharisma* exhibits considerable diversity in cell size and shape, with numerous morphospecies identified to date [5,6,8]. Our previous investigations and the present study shed light on the relationship between these morphospecies and biological species. Observations within these morphospecies revealed that gamone 1 does not function across different morphospecies, leading to reproductive isolation primarily attributed to gamone 1. Furthermore, the amino acid sequences of gamone 1 also vary across species boundaries. Even upon the artificial activation of cells, no interspecies conjugation pairs are formed between *B. undulans* (Mk2) and *B. japonicum* (Mk4) or between *B. undulans* (Mk2) and *B. americanum* (Mk3). In cases where interspecies conjugation pairs are artificially formed, such as between *B. americanum* (Mk3) and *B. stoltei* (Mk4), no offspring are produced; thus, reproductive isolation is established between these species. In essence, morphospecies of *Blepharisma* demonstrate a near correspondence to the biological species. In other words, variations in the amino acid sequences of gamone 1 reflect both morphospecies and biological species. For instance, in *B. americanum* and *B. japonicum*, distinct species-specific variations in the gamone 1 amino acid sequence were observed, resulting in a lack of gamone 1 interaction. These two species are morphologically distinct and also recognized as separate biological species.

As an exception, *B. japonicum* (Mk4) and *B. stoltei* (Mk4), despite representing different morphospecies, exhibit remarkably high similarity in their gamone 1 amino acid se-quences and can both activate gamone 1 in each other [23]. Classic reports of offspring between these two species support their classification as the same biological species [46]. Therefore, while they represent distinct morphospecies, they can be considered as a single biological species.

### 4.4. Morphospecies and Biological Species, and Speciation in Blepharisma

The phylogenetic relationships within *Blepharisma* have historically relied primarily on morphology and SSU rRNA gene analyses [5,6,7,8]. However, due to the lack of sequence variability across the species of *Blepharisma*, the SSU rRNA gene was deemed inadequate to elucidate species diversification processes and phylogenetic relationships. The investigation of amino acid sequences of the gamone 1 homologs in this study holds promise for unraveling the process of species diversification. The potential extension of these findings to numerous other species of *Blepharisma* awaits further investigation.

The intriguing question of why *Blepharisma* displays such diverse morphologies remains. *Blepharisma* exhibits significant morphological alterations based on environmental conditions; for instance, under starvation, they undergo predatory enlargement. Given the potential to adopt flexible morphologies of *Blepharisma* in response to environmental cues, this adaptability likely accounts for the emergence of diverse morphospecies. *Blepharisma* is known to inhabit extreme environments, such as hypersaline lagoons [3,4]. *Blepharisma* has adapted to such diverse environments, potentially giving rise to the emergence of new morphospecies. Among these, some species, such as *B. japonicum* and *B. stoltei*, represent distinct morphospecies while retaining high homology in gamone 1, allowing the completion of conjugation without achieving the status of biological species. Conversely, many cases have established themselves as distinct morphospecies and biological species. Future exploration, involving the examination of gamone 1 homologous gene amino acid sequences and morphology across a wider array of *Blepharisma* morphospecies, will likely provide deeper insights into *Blepharisma*’s speciation.

This study compared the amino acid sequences of gamone 1 among different species of *Blepharisma* and proposed the possibility that gamone 1 variations are a factor in species diversification.

## 5. Conclusions

The amino acid sequences of the mating pheromone, gamone 1, were compared across different morphospecies of *Blepharisma*, revealing distinct sites of amino acid sequence divergence across species boundaries. These variations appear to influence the species-specific gamone 1 function, suggesting their potential contribution as factors driving the diversification of species within the genus *Blepharisma*. In our future research, we aim to further investigate the gamone 1 gene in numerous species of *Blepharisma* to increase the evidence supporting the role of gamone 1 mutations in driving species diversification. Additionally, we intend to explore whether our hypotheses regarding species differentiation in *Blepharisma* are applicable to other ciliates.

## Figures and Tables

**Table 1:** Strains used in this study.

Mk	Species	Mating Type	Strain *	Long Diameter	Short Diameter	Collection Site
2	*B. undulans*	I	EN-II	137	60	Ibaraki, Japan
			SDT2-II	130	48	Minamidaito Island, Japan
			K78	119	49	Ishikawa, Japan
		II	SDT1-II	111	22	Minamidaito Island, Japan
3	*B. americanum*	I	CCAP1607/1 *	233	105	USA
			SM-III *	148	61	unidentified
		II	KR-III *	148	44	Gyeongju, South Korea
	*B. musculus*	I	SDT0-III *	166	64	Minamidaito Island, Japan
			SG1-III *	145	58	Ibaraki, Japan
4	*B. japonicum*	I	R1072	340	164	Bangalore, India
		II	YC-IV	232	104	Yamaguchi Japan
			KG-IV *	251	103	Nara, Japan
	*B. stoltei*	I	ATCC30299	211	86	Lake Federsee, Germany
		II	HT-IV	199	75	Aichi, Japan
			WK-IV *	176	62	Hokkaido, Japan

Mk: megakaryotype [10]. Cell dimensions (long and short diameters) are shown as averages (µm). * Strains in which gamone 1 homologs were newly isolated and sequenced in this study.

**Table 2:** Gamone 1 homologies in 15 strains.

			Megakaryotype 2			Megakaryotype 3					Megakaryotype 4					
			*B. undulans*			*B. americanum*			*B. musculus*		*B. japonicum*			*B. stoltei*		
			K78	SDT2-II	SDT1-II	CCAP 1607/1	SM-III	KR-III	SG1-III	SDT0-III	R1072	KG-IV	YC-IV	ATCC 30299	HT-IV	WK-IV
Mk2	*B. undulans*	EN-II	95.7	95.7	96.1	73.1	73.1	72.8	73.7	73.4	73.1	75.1	75.1	75.1	74.4	74.8
		K78	-	98.7	99.7	73.4	73.4	73.1	72.4	72.0	73.1	75.1	75.1	75.1	74.4	74.8
		SDT2-II	-	-	99.0	74.1	74.1	73.8	73.0	72.7	74.1	76.1	76.1	76.1	75.4	75.7
		SDT1-II	-	-	-	73.8	73.8	73.4	72.7	72.4	73.4	75.4	75.4	75.4	74.8	75.1
Mk3	*B. americanum*	CCAP1607/1	-	-	-	-	100	99.7	70.4	70.1	72.1	72.1	72.1	72.8	72.1	72.5
		SM-III	-	-	-	-	-	99.7	70.4	70.1	72.1	72.1	72.1	72.8	72.1	72.5
		KR-III	-	-	-	-	-	-	70.4	70.1	72.1	72.1	72.1	72.8	72.1	72.5
	*B. musculus*	SG1-III	-	-	-	-	-	-	-	99.7	68.1	69.1	69.1	69.7	69.1	68.8
		SDT0-III	-	-	-	-	-	-	-	-	67.8	68.8	68.8	69.4	68.8	68.4
Mk4	*B. japonicum*	R1072	-	-	-	-	-	-	-	-	-	88.2	88.2	87.5	88.2	88.5
		KG-IV	-	-	-	-	-	-	-	-	-	-	100	95.7	99.3	99.7
		YC-IV	-	-	-	-	-	-	-	-	-	-	-	95.7	99.3	99.7
	*B. stoltei*	ATCC30299	-	-	-	-	-	-	-	-	-	-	-	-	95.7	95.4
		HT-IV	-	-	-	-	-	-	-	-	-	-	-	-	-	99.0

Mk: megakaryotype. Homology (%) was calculated using ClustalW. A cell with homology of 87% or higher was colored gray.

## Data Availability

The data of this study are available from the corresponding author upon reasonable request.
